# Synergism of 2-(3,4-dichlorophenoxy)triethylamine and top removal enhances maize lodging resistance and yield via coordinated photosynthetic optimization and phenylpropanoid pathway activation

**DOI:** 10.3389/fpls.2025.1671417

**Published:** 2025-12-15

**Authors:** Tenglong Xie, Meiyu Wang, Xiaoge Yang, Yihe Ma, Baoyu Zhang, Qian Zhang, Linlin Mei, Deguang Yang

**Affiliations:** 1 Northeast Agricultural University, Harbin, China; 2 Agricultural Officer, Amur Town People’s Government, Mohe, China

**Keywords:** maize, lodging resistance lodging stress, top removal, DCPTA, photosynthesis, phenylpropanoid pathway

## Abstract

**Introduction:**

Plant growth promoters like 2-(3,4-dichlorophenoxy) triethylamine (DCPTA) increases maize yield but heightens lodging risk. We investigated whether integrating canopy top removal (TR) with DCPTA application resolves this trade-off.

**Methods:**

Over 2023–2024 seasons, maize plants were treated with DCPTA, TR, or both. Assessments included: the basal 8th leaf photosynthesis; strength, phenylpropanoid enzyme activity, and structural components deposition in the basal 3rd internode; plus grain yield and lodging incidence.

**Results:**

TR counteracted DCPTA-induced suppression of basal leaf photosynthesis and carbon assimilation. Crucially, the combination synergistically enhanced assimilate flux to basal stalks. This influx, potentiated by DCPTA, significantly upregulated phenylpropanoid enzymes versus TR alone, driving enhanced deposition of structural components. Specifically, during the grain-filling stage, the combination significantly enhanced chlorophyll content, gas exchange parameters (P_n_ and G_s_), and PSII photochemical efficiency (Fv/Fm, ΦPSII, and ETR) in the basal 8th leaf, while reducing NPQ, thereby optimizing overall photosynthetic performance. Consequently, stem strength markedly increased. Over 2023–2024 growing seasons, the integrated strategy consistently increased grain yield: the TR+DCPTA treatment increased grain yield by 5.64%/4.56% vs. DCPTA alone, 3.45%/3.69% vs. TR alone, and 9.34%/8.19% vs. the control, respectively. Meanwhile, lodging incidence dropped significantly by 81.25% (2023) and 70.18% (2024) compared with sole DCPTA application.

**Discussion:**

The synergy arises because TR alleviates DCPTA’s negative effects on basal leaf function, while DCPTA potentiates the phenylpropanoid pathway’s response to the assimilate surge triggered by TR. This reciprocal interaction enables simultaneous optimization of photosynthetic efficiency in source leaves and robust activation of stem strengthening mechanisms, achieving higher yield and lodging resistance.

## 1 Introduction

Maize (*Zea mays* L.) is a critical global cereal crop, facing escalating demand driven by population growth and livestock feed requirements (FAOSTAT, 2023). Enhancing yield potential while ensuring stability is paramount for sustainable production (Luo et al., 2023). Plant growth promoters, such as glutamine (Islam et al., 2024), melatonin (Xu S, et al., 2024), and 6-benzyladenine (Gao et al., 2017), can boost maize growth and yield potential. However, their application often induces excessive stem elongation, heightening lodging susceptibility and negating yield gains under suboptimal conditions (Li et al., 2019). Yield losses correlate strongly with lodging incidence, estimated at 6-8% per 10% increase in lodging rate. The severity of lodging varies among different growth promoters—it is not uniform. Against this backdrop, the selection of DCPTA in this experiment does not negate the value of other growth promoters; instead, it is based on its direct enhancement of photosynthetic efficiency, systematic regulation of stress resistance, and economic viability in application (Qu et al., 2014).

To reduce lodging vulnerability, extensive research is centered on the combined application of plant growth promoters and growth-retarding compounds like ethephon (Zhang et al., 2014) or chlorocholine chloride (Wang et al., 2016). Nevertheless, this approach involves a fundamental physiological compromise. Although growth-retarding compounds can effectively improve the mechanical strength of stems and lodging resistance, their use often disturbs photosynthetic efficiency and assimilate partitioning. As a result, inappropriate application regimens or environmental interactions carry a significant risk of inadvertently suppressing yields (Makleit et al., 2024).

Strategic canopy restructuring through the targeted removal of top organs (tassel and/or top leaves) during the grain-filling stage is a valuable agronomic method for optimizing maize productivity (Gao et al., 2020). Specifically, top removal, which focuses on the uppermost canopy layers, significantly improves the light microenvironment within the canopy. This enhancement promotes higher photosynthetic efficiency in the crucial ear leaves (Liu et al., 2015) and simultaneously stimulates the root nutrient acquisition ability (Raza et al., 2020). Moreover, top removal substantially increases the mechanical strength and lodging resistance of the basal culm internodes by increasing the deposition of structural carbohydrates (Xue et al., 2017). Importantly, the existence of mature mechanized top-removal equipment makes implementing this canopy management strategy both feasible and scalable under current maize field conditions.

Studies on the combination of chemical regulation and physical canopy reconstruction have been reported. For example, the combination of uniconazole and shoot tip pruning can optimize flowering induction and fruit quality of mangoes (Oliveira et al., 2017), while a single plant growth regulator can modulate the canopy structure of maize to reduce lodging rate (Xie et al., 2019a; 2025). However, systematic research on the interaction between novel growth promoters centered on “enhancing photosynthetic efficiency” (such as DCPTA) and “canopy regulation via top removal”—including their synergistic strengthening effect on stem mechanical strength, the regulatory mechanism of DCPTA on photosynthetic compensation after top removal, and the dynamic balance of source-sink carbon allocation—has not been documented yet. The technical effects and action mechanism of this specific combination in high-density maize still remain a research gap. We postulated that top removal could counteract these negative effects while synergizing with DCPTA’s positive attributes.

This study investigated the physiological and metabolic basis for enhanced lodging resistance and yield in maize through the integrated application of DCPTA and top removal. Specifically, we aimed to: (1) Determine if top removal mitigates DCPTA-induced suppression of basal internode biomechanical strength while sustaining yield potential; (2) Elucidate the underlying mechanisms by examining: a. photosynthetic carbon assimilation and regulation in the pivotal 8th leaf (basal source); b. phenylpropanoid pathway (monomer supply, polymerization, deposition) dynamics in the load-bearing 3rd basal internode (the third internode above ground level). This is because the 8th leaf serves as the exclusive photosynthetic source for the key basal structural sink and exhibits functional stability, while the 3rd basal internode acts as the core structural sink that directly correlates with lodging rate (Liu et al., 2015; Kamran et al., 2018).

## 2 Materials and methods

### 2.1 Experimental site, design and sampling

Field experiments were conducted during the maize growing seasons (May-October) of 2023 and 2024 at the Experimental Station (45°46′N, 126°54′E) of the Northeast Agricultural University, Harbin City, Heilongjiang Province, China. We used the widely adapted hybrid “Zhengdan 958” (provided by Henan Academy of Agricultural Sciences). We sourced DCPTA (with a purity of ≥95%) from Zhengzhou Zhengshi Chemical Ltd. The experimental site was equipped with a drainage system. We conducted deep plowing before sowing and carried out timely hilling during the growth period. These comprehensive measures effectively facilitated root elongation and sturdy development, significantly improving the soil anchorage ability and lodging resistance. As a result, we seldom observed root lodging in this experiment.

A randomized complete block design (RCBD) was adopted, with 4 treatments and 5 replicates per treatment:

(1) CK (Control): No DCPTA application or top removal;

(2) DCPTA: Foliar spray of 35 mg·L^−1^ DCPTA (supplemented with 0.03% Tween-20 as surfactant) at 450 L·ha^−1^ at the 8-leaf stage (V8).

The V8 stage was selected because the V8–V12 period (internode lignification stage) is critical for lodging resistance establishment. Applying DCPTA at V8 maximizes its functions in regulating stalk development and enhancing photosynthesis, while avoiding growth cycle disruption to balance lodging resistance and yield improvement;

Note: For spraying, ensure meteorological conditions are met (temperature: 20–25°C, relative humidity: 60%–70%, wind speed < 2 m/s) and apply uniformly over the entire plant, with the solution adhering to both leaf surfaces, to guarantee experimental rigor;

(3) TR (Top Removal): Manual removal of tassels and two uppermost leaves post-ear pollination.

Cuts were made at the leaf collar (annular structure at the leaf blade-sheath junction), with 2–3 cm of leaf sheath retained; cuts were ensured smooth and free of tears or fraying;

(4) Combined treatment of DCPTA (as described in Treatment 2) and TR (as described in Treatment 3).

Each plot had an area of 1000 m² (38.46 m × 26.00 m), consisting of 40 rows with a row spacing of 0.65 m. Pre-sowing soil chemical properties were analyzed following the method of Cottenie et al. (1982), and results are presented in **Table 1**.

**Table 1:** Chemical properties of the used soil.

Year	pH	HCO_3_^-^+ CO_3_^2-^	Cl^-^	SO_4_^2-^	Ca^2+^	Mg^2+^	Na^+^	K^+^	N	P
2023	7.2	204.5	293.8	447.3	89.7	39.8	4.1	29.4	16.8	3.7
2024	7.3	207.1	305.2	452.9	92.5	42.3	4.3	32.2	17.2	3.9

Seeds of the maize hybrid “Zhengdan 958” were hand-sown at a density of 7 plants·m^−2^ on May 5 (2023) and May 2 (2024). A slow-release compound fertilizer (25% N, 10% P_2_O_5_, 13% K_2_O) was uniformly applied at 700 kg·ha^−1^ (equivalent to 70 kg per plot), without adjustment based on baseline soil fertility. Standard agronomic practices (weed, pest, and disease control) were consistent across all plots.

**Figure 1:**
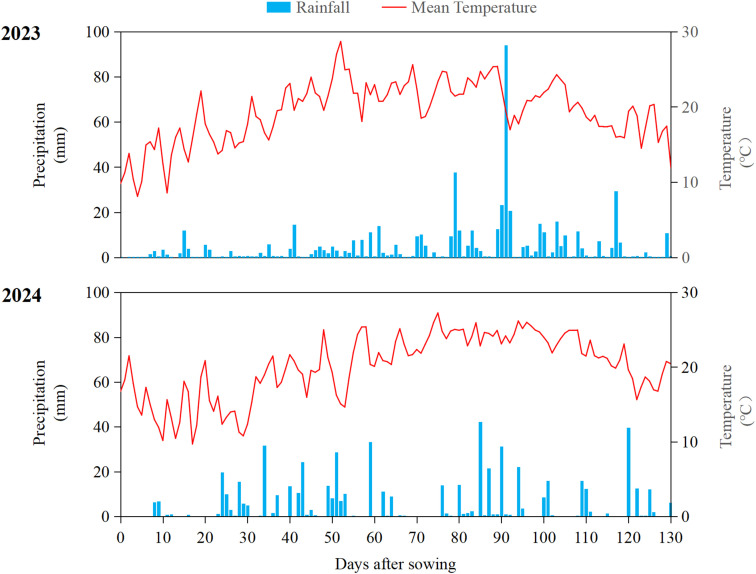
The rainfall (bar) and mean temperature (line) data during the study period (2023 and 2024, May–October).

Harvest was conducted on October 7 (2023) and October 10 (2024). Meteorological data (rainfall and mean temperature) during the growing seasons (May-October 2023-2024) are shown in **Figure 1**. Extreme weather (sudden heavy rainfall and sharp temperature drop) occurred in August 2023, while 2024 had favorable and stable conditions. No significant difference in stalk lodging patterns was observed between the two years, indicating that the 2023 extreme weather did not affect lodging-related observations.

Destructive sampling was performed at six key growth stages: R1 (silking), R2 (blister), R3 (milk), R4 (dough), R5 (dent), and R6 (physiological maturity). Independent plant sets were used at each stage to avoid interference from prior measurements.

8th leaf: Collected for the determination of photosynthetic parameters (gas exchange, chlorophyll fluorescence). The central midrib and 2–3 mm regions on both sides of the midrib were avoided, and the upper-middle region of the leaf was prioritized (Liu et al., 2014; Xue et al., 2017);

3rd basal internode: Collected adjacent to the 8th leaf for analyses of stalk morphology, mechanical strength, and physiology.

Samples were selected based on temporal-spatial consistency, physiological representativeness, and freedom from interference.

### 2.2 Yield components and lodging rate

We measured the grain yield at the R6 stage from the central 15 rows (with a length of 10.26 m), and assessed the internode lodging rate for the entire plot. Internode lodging was defined as stalk bending with an angle ≥45°from the vertical axis or stalk fracture occurring at the 3rd basal internode (Li et al., 2021). We recorded the number of effective harvestable ears per hectare (ears ha^−1^), the number of kernels per ear (kernels ear^−1^), the 1000-kernel weight, and the number of internode lodging plants; these parameters were then calculated using the following formula;


$$\text{Grain yield }({\text{kg ha}}^{-1})=[{\text{Effective harvestable ears ha}}^{-1}\times {\text{kernels ear}}^{-1}\times 1,000-\text{kernel weight }(\text{g})]/[(1–\text{Moisture content}\%)\times (1–14\%)]$$

(Song et al., 2024);


Internode lodging rate (%)=(Number of internode lodging plants/Total plants per plot)×100

(Li et al., 2021).

Among them, the grain moisture content was determined using a portable grain moisture meter (Model: PM-8188) on the grains harvested at the R6 stage, and “14%” refers to the standard moisture content used for grain yield calculation.

### 2.3 Internode morphology

At the R3 growth stage, 10 fresh plants with typical and uniform morphology were selected from each plot referenced from Xue et al. (2017). We recorded plant height as the vertical stature of the main stem. For the third internode, we determined its length between adjacent nodes. We assessed internode diameter—defined as the average of the minor and major axes measured at the midpoint—using a Vernier caliper.

The R3 stage was chosen because it marks the completion of 3rd basal internode elongation (Xue et al., 2017) and the initiation of structural carbohydrate accumulation—its morphological traits are stably formed at this stage, avoiding data bias from tissue senescence and dehydration that occur at the R6 stage.

### 2.4 Internode mechanical properties

Select disease-free plants with uniform internode thickness for sampling. Mechanical strength parameters for the third internode above ground level were quantified using an AWOS-SL04 stalk strength tester, focusing on rind penetrating strength (*PS*) and bending strength (*BS*).

*PS* Measurement: Firmly fix the internode, and the puncture point should be at the midpoint of the internode. A needle probe (1 cm long, 1 mm² cross-section) was driven perpendicularly into the stalk at a controlled rate until the stop bar contacted the surface. The peak force registered was recorded as PS.

*BS* Measurement: For the bending test, place support points 2 cm from each end of the internode, and position the support plates at this spacing (i.e., 10 cm apart). A vertically aligned U-shaped probe was centered on the internode midpoint and steadily depressed until structural failure occurred. The maximum force sustained, displayed as BS (in Newtons), was captured. All strength assessments targeted the widest face of the internode midpoint.

### 2.5 Carbohydrate composition

Following mechanical strength and plumpness evaluations (methodology detailed subsequently), the third internodes were excised at their nodal junctions. Segments for analysis were prepared by removing 0.5 cm sections from both proximal and distal internode ends. We analyzed the soluble sugar concentration via the anthrone-sulfuric acid colorimetric procedure. Samples underwent drying at 80°C in a forced-air oven until constant mass was achieved for dry weight determination. Internode plumpness, defined as dry mass per unit length (mg cm^-1^), was computed as:


Plumpness=Internode dry mass (mg)/Internode length (cm)

(Ahmad et al., 2021)

The desiccated internode segments were pulverized using a laboratory mill, passed through a 0.5-mm sieve, and the resultant flour was desiccated and stored at 4°C pending compositional assays. Lignin content was analyzed using the protocol of Peng et al. (2014); cellulose quantification employed anthrone colorimetry (Zhang et al., 2020); hemicellulose levels were determined according to Li et al. (2016).

### 2.6 Phenylpropanoid pathway enzyme activities

Fresh 4-cm mid-sections from the third internode were immediately flash-frozen in liquid nitrogen and stored at -80°C. Enzyme activities for phenylalanine ammonia-lyase (PAL), 4-coumarate: CoA ligase (4-CL), and cinnamyl alcohol dehydrogenase (CAD) were quantified using commercial ELISA kits (Suzhou Geruisi Biotechnology, Jiangsu, China). Peroxidase (POD) activity was assayed based on the method by Moerschbacher et al. (1988).

### 2.7 Canopy structure

The leaf area index (LAI) and photosynthetically active radiation (PAR) of each plot were measured at R3. LAI was calculated based on the plant density and the total leaf area per plant (computed using the formula: A = 0.75 × L × W). PAR was measured above the 8th leaves using a SpectraPen LM500 hand-held spectrometer (Photon Systems Instruments, Drásov, Czechia) on a sunny and cloudless day according to Yan et al. (2024).

### 2.8 Chlorophyll content

Leaf chlorophyll was isolated by homogenizing tissue samples in 10 mL of 80% (v/v) acetone. Following centrifugation at 1000 × g (4°C, 3 min), the pellet was resuspended in a fresh 10 mL aliquot of 80% aqueous acetone and subjected to a 24-hour dark incubation at ambient temperature. The 24-hour dark incubation was validated to reduce impurity interference by 13%-16% and limit chlorophyll degradation to <3% (Pedraza-González et al., 2024), ensuring extraction accuracy for basal leaves with high phenolic content.

Post-incubation, the clarified supernatant was collected and its absorbance measured at 652 nm using a SpectraMax i3x microplate reader (Molecular Devices). A wavelength of 652 nm is the “cross wavelength” between the absorption peaks of chlorophyll a (663 nm) and chlorophyll b (645 nm), and it is used for the rapid determination of total chlorophyll content. Chlorophyll concentration (mg g^-1^ fresh weight) was subsequently calculated based on the absorbance reading.

### 2.9 Chlorophyll fluorescence parameters

Chlorophyll fluorescence parameters were assessed using a LI-6400–40 integrated leaf chamber fluorometer (LI-COR Biosciences, Lincoln, NE, USA) coupled to a portable LI-6400XT photosynthesis system (LI-COR), following the methodology outlined by Maxwell and Johnson (2000). Prior to measurement, the 8th leaves underwent dark adaptation for over 30 minutes. In the dark-adapted state, the maximum quantum efficiency of photosystem II (*Fv/Fm*) was quantified by applying a saturating light pulse (PAR>6000 μmol m^-2^ s^-1^, duration 0.8 s). Subsequently, leaves were illuminated with actinic light matching the experimental photosynthetic photon flux density (*PPFD*). Once steady-state photosynthesis was achieved, parameters including the effective quantum yield of *PSII* (*ΦPSII*), non-photochemical quenching (*NPQ*), and the estimated electron transport rate (*ETR*) were determined using the saturation pulse technique.

### 2.10 Gas exchange parameters

Measurements of net photosynthesis (*Pn*), transpiration rate (*Tr*), stomatal conductance (*Gs*), and intercellular CO_2_ concentration (*Ci*) were conducted from 10:00 to 14:00 h using an LI-6400XT photosynthesis system. Key operating parameters were: 500 μmol s^-1^ flow rate, 1500 μmol m^-2^ s^-1^ incident PPFD, and 400 μmol mol^-1^ reference [CO_2_]. Leaf chamber area was ~6 cm², and temperature was maintained at ~26°C (Tollenaar, 1999).

### 2.11 Carbon metabolism enzyme activities

The activities of ribulose-1,5-bisphosphate carboxylase/oxygenase (Rubisco) and phosphoenolpyruvate carboxylase (PEPC) were measured according to Xie et al. (2017). The specific methods are as follows:

Fresh leaf samples (0.5 g) from the 8th leaf were homogenized in an ice-cold extraction buffer (50 mM HEPES-KOH, pH 7.8, containing 10 mM MgCl_2_, 1 mM EDTA, 5 mM DTT, and 1% PVP). The homogenate was centrifuged at 12,000 × g for 15 min at 4°C, and the supernatant was used as the enzyme extract.

Rubisco activity was assayed by monitoring NADH oxidation at 340 nm in a reaction mixture initiated with ribulose-1,5-bisphosphate. PEPC activity was determined by coupling the reaction to malate dehydrogenase and measuring NADH oxidation. Enzyme activities were expressed as μmol NADH min^−1^ mg^−1^ protein, with protein content determined by the Bradford method.

### 2.12 Statistical analysis

One-way analysis of variance (ANOVA) was performed using SPSS software (version 18.0) to evaluate the effects of different treatments (CK, DCPTA, TR, TR+DCPTA) on each measured index. In this study, “treatment” was defined as the sole fixed factor, while “growth stage” served merely as a temporal observation node rather than an interactive factor. A P-value < 0.05 was considered to indicate a statistically significant difference in the index among various treatment groups.

## 3 Results

### 3.1 Yield components and lodging resistance

Analysis of the two-year field data revealed significant treatment effects on maize yield components and lodging resistance (**Table 2**). Compared to the control (CK), DCPTA alone significantly increased kernel weight and grain yield (+5.69% to 11,138 kg ha^-1^ in 2023; +4.55% to 10,763 kg ha^-1^ in 2024; P < 0.05), but concomitantly induced severe lodging (representing increases of 69.36% and 91.10% over CK). Conversely, TR alone effectively suppressed lodging, whereas it yielded less than DCPTA (10,908.00 kg ha^-1^ and 10,674.00 kg ha^-1^ in 2023 and 2024, respectively) due to reduced kernel weight. Critically, the TR+DCPTA combination resolved these antagonisms, achieving the highest yields (11,522 kg ha^-1^ in 2023 and 11,137 kg ha^-1^ in 2024). These yields exceeded CK by 9.34% and 8.19%, DCPTA alone by 5.64% and 4.56%, and TR alone by 3.45% and 3.69% in 2023 and 2024, respectively. This synergistic effect stemmed from optimizing kernel number per ear and enhancing kernel weight, coupled with an increased harvestable ear number (exceeding DCPTA by 4.30% and 4.66% in 2023 and 2024, respectively) attributable to minimal lodging – reduced by 81.25% and 70.18% compared to DCPTA alone.

**Table 2:** Effects of DCPTA and/or top removal on maize yield components and lodging resistance across two growing seasons (2023-2024).

Years	Treatment	Number of ears (ears ha–1)	Number of kernels (kernels ear–1)	1000-kernel weight (g)	Yield (kg ha–1)	Internode lodging rate (%)
2023	CK	67920 ± 497b	511 ± 4b	303.74 ± 1.47d	10538 ± 110d	2.97% ± 0.71%b
	DCPTA	66480 ± 907c	514 ± 6ab	325.98 ± 2.78a	11138 ± 123b	5.03% ± 1.30%a
	TR	68880 ± 432a	513 ± 4b	308.51 ± 0.86c	10908 ± 87c	1.60% ± 0.62%c
	TR+DCPTA	69340 ± 611a	521 ± 8a	318.74 ± 2.92b	11522 ± 108a	0.94% ± 0.87%c
2024	CK	67720 ± 567b	506 ± 6b	300.63 ± 1.77d	10294 ± 141c	3.26% ± 0.81%b
	DCPTA	65640 ± 416c	511 ± 5ab	321.18 ± 2.36a	10763 ± 105b	6.23% ± 0.59%a
	TR	68200 ± 663ab	512 ± 5ab	305.64 ± 1.32c	10674 ± 62b	2.57% ± 0.95%bc
	TR+DCPTA	68700 ± 815a	517 ± 5a	313.81 ± 1.79b	11137 ± 128a	1.86% ± 1.16%c

*The values represent the means ± SEs (n=5). The values with the same letters in the columns are not significantly different at P<0.05 (LSD test).

### 3.2 Agronomic traits

Quantification of maize morpho-physiological responses over the 2023–2024 seasons revealed distinct treatment effects (**Table 3**). DCPTA alone significantly increased plant height (+7.39% and +9.02%) and LAI (+5.18% and +4.65%), decreased PAR (-9.42% and -8.69%) relative to CK. This was accompanied by elongation of the 3rd basal internode (+12.32% and +15.06%), but a reduction in its diameter (-14.13% and -13.52%). In contrast, TR alone decreased plant height (-11.28% and -7.59%) and LAI (-5.21% and -7.49%), while elevating PAR (+5.68% and +6.25%) without altering internode metrics. The DCPTA+TR interaction yielded intermediate phenotypes: internode elongation persisted (+11.24% and +14.91%) with reduced diameter (-8.26% and -6.91%), and plant height remained suppressed (-7.04% and -3.98%). Notably, although LAI did not differ significantly from CK, PAR increased substantially (+1.79% and +3.48%).

**Table 3:** Effects of DCPTA and/or top removal on maize Agronomic traits across two growing seasons (2023-2024).

Years	Treatment	Plant height (cm)	3rd basal internode		LAI	PAR on 8th leaf (μmol m^-2^ s^-1^)
			Length (cm)	Diameter (cm)		
2023	CK	273.83 ± 5.81b	11.71 ± 0.91b	29.25 ± 1.29a	5.49 ± 0.17b	158.91 ± 6.36 b
	DCPTA	294.08 ± 4.52a	13.16 ± 0.69a	25.12 ± 1.96b	5.78 ± 0.11a	143.94 ± 4.13 c
	TR	242.94 ± 4.38d	11.85 ± 0.86b	29.40 ± 1.62a	5.21 ± 0.23c	167.93 ± 7.02 a
	TR+DCPTA	254.54 ± 7.63c	13.03 ± 0.76a	26.83 ± 1.53b	5.46 ± 0.16b	161.75 ± 6.15 ab
2024	CK	253.70 ± 3.95b	10.84 ± 0.56b	25.29 ± 1.59a	5.57 ± 0.27b	151.06 ± 7.11 a
	DCPTA	276.58 ± 6.24a	12.47 ± 0.89a	21.87 ± 1.43b	5.83 ± 0.15a	137.94 ± 8.62 b
	TR	234.45 ± 9.56c	10.93 ± 0.64b	25.62 ± 1.22a	5.15 ± 0.14c	160.49 ± 7.91 a
	TR+DCPTA	243.61 ± 8.43c	12.34 ± 0.50a	23.54 ± 1.36b	5.640.17ab	156.31 ± 8.24 a

*The values represent the means ± SEs (n=5). The values with the same letters in the columns are not significantly different at P<0.05 (LSD test).

### 3.3 Temporal dynamics of the 3rd internode mechanical strength

**Figure 2:**
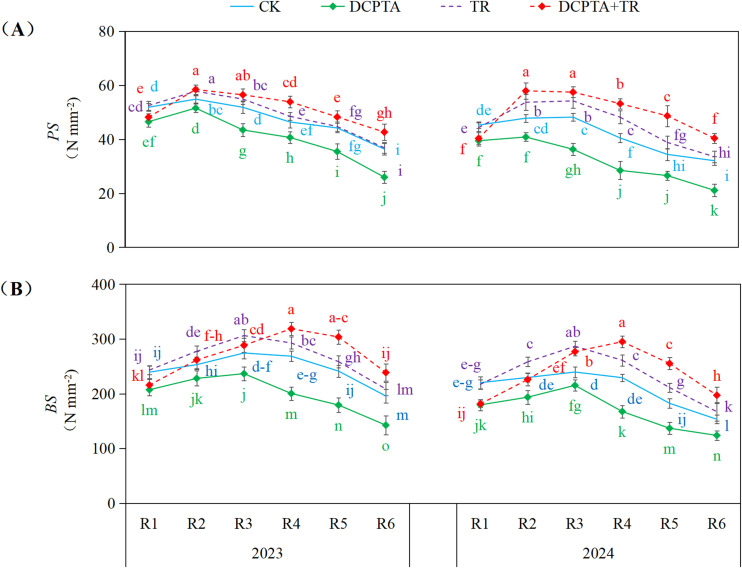
Effects of DCPTA and/or top removal on 3rd internode mechanical strength across two growing seasons (2023-2024). **(A)** represents rind penetrating strength (PS); **(B)** represents bending strength (BS). Lowercase letters (a, b, c...) indicate significant differences among treatments (P<0.05, LSD test).

DCPTA pretreatment alone consistently impaired internode mechanical strength across both years, significantly reducing *PS* and *BS* (e.g., 2024 R6 PS: -34.25%; 2024 R4 BS: -26.98%) (**Figure 2**). Conversely, TR alone generally enhanced strength, particularly during later reproductive stages (e.g., 2024 R4 PS: +18.95%; 2024 R3 BS: +19.67%). Remarkably, combining DCPTA with TR triggered potent synergy, not only overcoming the suppressive effect of DCPTA but also amplifying the benefits of TR. This synergistic enhancement intensified progressively from stages R4 to R6, culminating in substantial increases by 2024 (e.g., R5 PS: +41.47%; R4 BS: +28.70%; R5 BS: +39.87%).

### 3.4 Metabolic reprogramming of the 3rd internode carbohydrates

**Figure 3:**
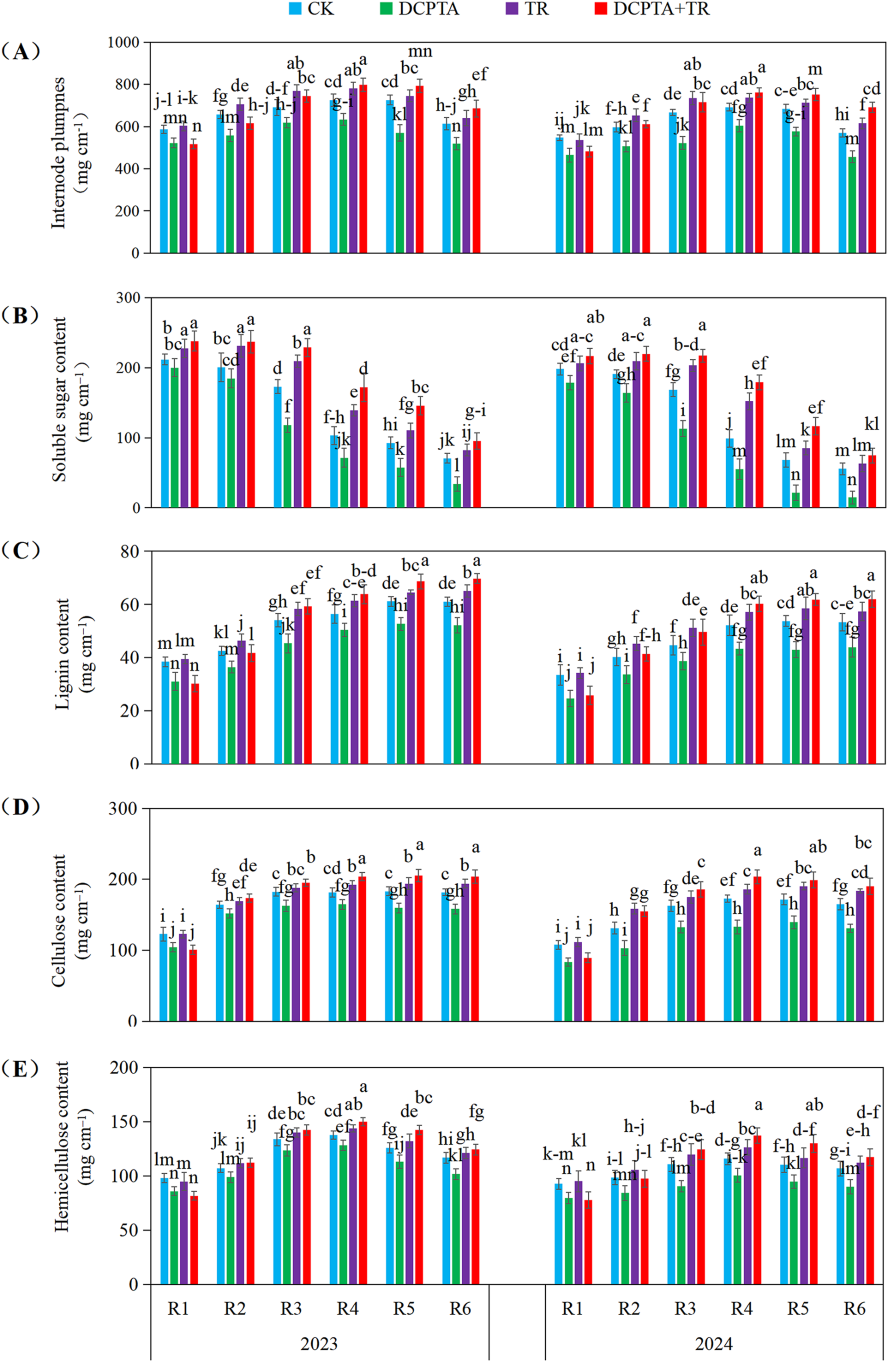
Effects of DCPTA and/or top removal on contents of the 3rd internode carbohydrates across two growing seasons (2023-2024). **(A)** Represents internode plumpness; **(B)** Represents soluble sugar content; **(C)** Represents lignin content; **(D)** Represents cellulose content; **(E)** Represents hemicellulose content. Lowercase letters (a, b, c...) indicate significant differences among treatments (P<0.05, LSD test).

Analysis revealed stage-dependent treatment effects on internode carbohydrate physiology relative to CK (**Figure 3**). DCPTA pretreatment alone consistently exerted negative effects on all parameters (plumpness, soluble sugars, lignin, cellulose, hemicellulose) across all stages (R1-R6) in both years, with soluble sugar depletion being particularly severe (e.g., 2024 R6: -73.71%). TR alone, however, generally enhanced most parameters, significantly boosting soluble sugars (e.g., 2023 R4: +35.02%). The combined treatment (DCPTA+TR) exhibited a dynamic interaction: suppressive or neutral in early stages (R1-R2), it shifted to pronounced synergistic enhancement in later stages (R3-R6). This synergy was most dramatic for soluble sugars (e.g., 2024 R4: +81.09%; 2024 R5: +69.82%) and lignin content (e.g., 2024 R6: +16.32%), effectively countering the DCPTA-induced deficits and frequently exceeding the benefits of TR alone, particularly for carbohydrate components from R3 onwards. This synergistic pattern was highly consistent across both years.

### 3.5 Synergistic activation of the phenylpropanoid pathway

**Figure 4:**
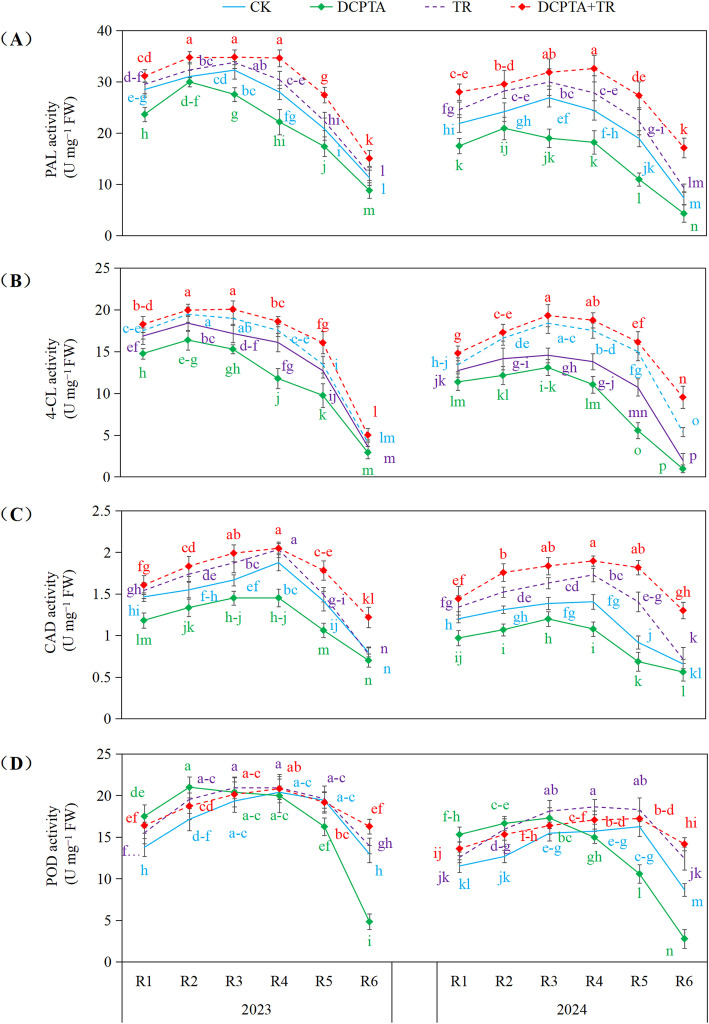
Effects of DCPTA and/or top removal on activities of 3rd internode phenylpropanoid pathway enzymes across two growing seasons (2023-2024). **(A)** Represents phenylalanine ammonia-lyase (PAL) activity; **(B)** Represents 4-coumarate: CoA ligase (4-CL) activity; **(C)** Represents cinnamyl alcohol dehydrogenase (CAD) activity; **(D)** Represents peroxidase (POD) activity. Lowercase letters (a, b, c...) indicate significant differences among treatments (P<0.05, LSD test).

Application of the DCPTA+TR combination elicited a marked synergistic activation of key phenylpropanoid pathway enzymes (PAL, 4-CL, CAD, POD) across developmental stages (R1-R6) in both experimental years (**Figure 4**). Pretreatment with DCPTA alone consistently suppressed enzyme activity relative to the control (CK), yielding negative changes for PAL, 4-CL, and CAD at nearly all stages, and for POD beyond R4 in 2023 and R3 in 2024. TR application alone induced moderate increases. Crucially, the combined treatment dramatically amplified enzyme activation, particularly during stages R4-R6. This synergy was exceptionally pronounced for 4-CL and CAD in 2024, culminating in substantial R6 increases (4-CL: +378.16%; CAD: +97.68%). PAL activity also exhibited robust positive synergy (e.g., 2023 R5: +31.74%; 2023 R6: +33.29%). While DCPTA alone severely inhibited POD activity during later reproductive stages, the combined treatment substantially counteracted this suppression, even inducing strong increases (e.g., 2023 R6: +25.63%; 2024 R6: +63.18%). Collectively, these data demonstrate that TR reverses the inhibitory effect of DCPTA, transforming it into a potent activator of phenylpropanoid pathway enzymes.

### 3.6 Chlorophyll content

**Figure 5:**
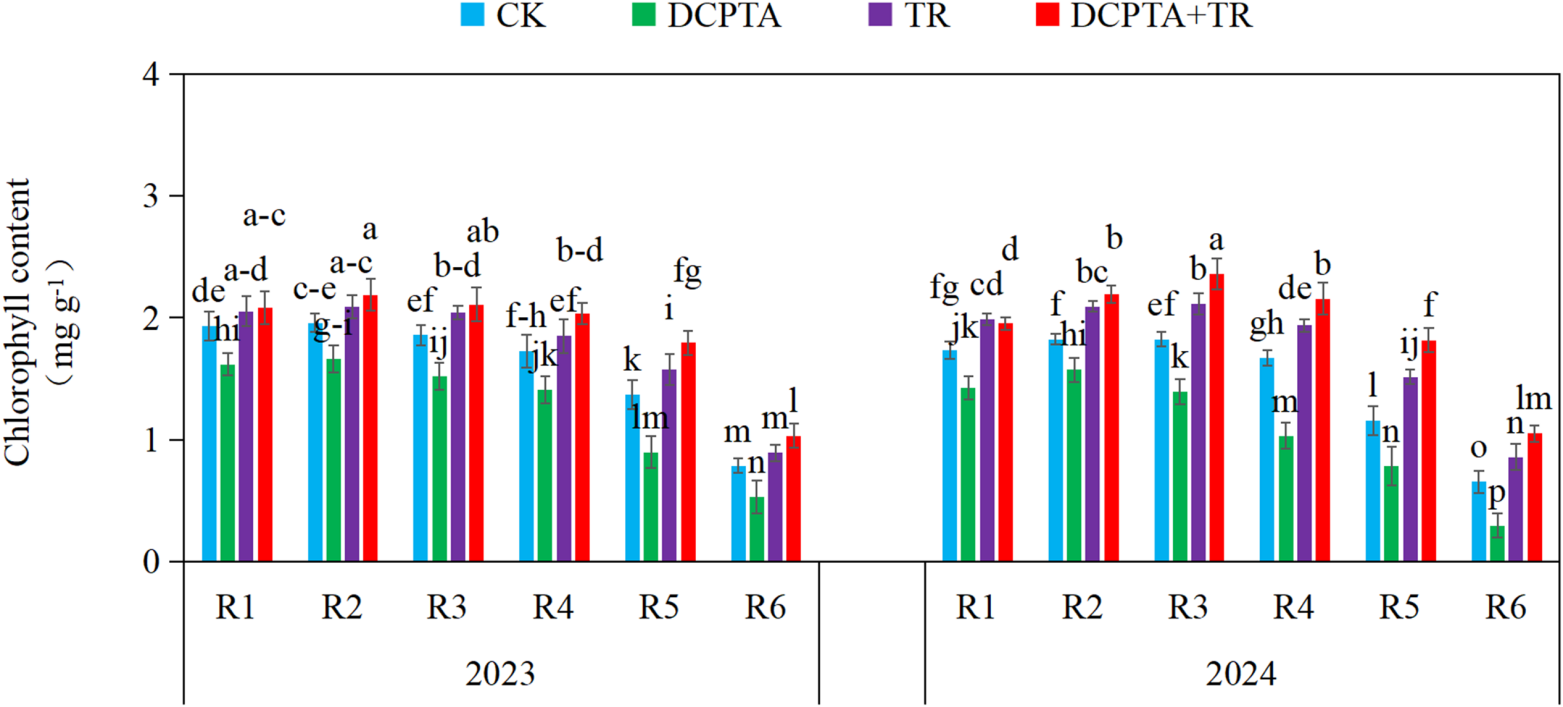
Effects of DCPTA and/or top removal on chlorophyll content of the 8th leaves across two growing seasons (2023-2024). Lowercase letters (a, b, c...) indicate significant differences among treatments (P<0.05, LSD test).

DCPTA pretreatment alone consistently reduced chlorophyll content across all stages (R1-R6) in both years, with suppression intensifying over time (peak suppression 2024 R6: -54.90%; **Figure 5**). In contrast, TR application alone universally increased chlorophyll content (+6.20% to +31.25%), with effects strengthening at later stages and in 2024. The combined DCPTA+TR treatment produced potent synergistic increases, significantly exceeding the effects of TR alone. These enhancements were particularly pronounced during late stages (R4-R6) in 2024, culminating in increases of +57.34% (R5) and +60.83% (R6). This potentiating effect became increasingly dominant throughout reproductive development.

### 3.7 Chlorophyll fluorescence parameters

**Figure 6:**
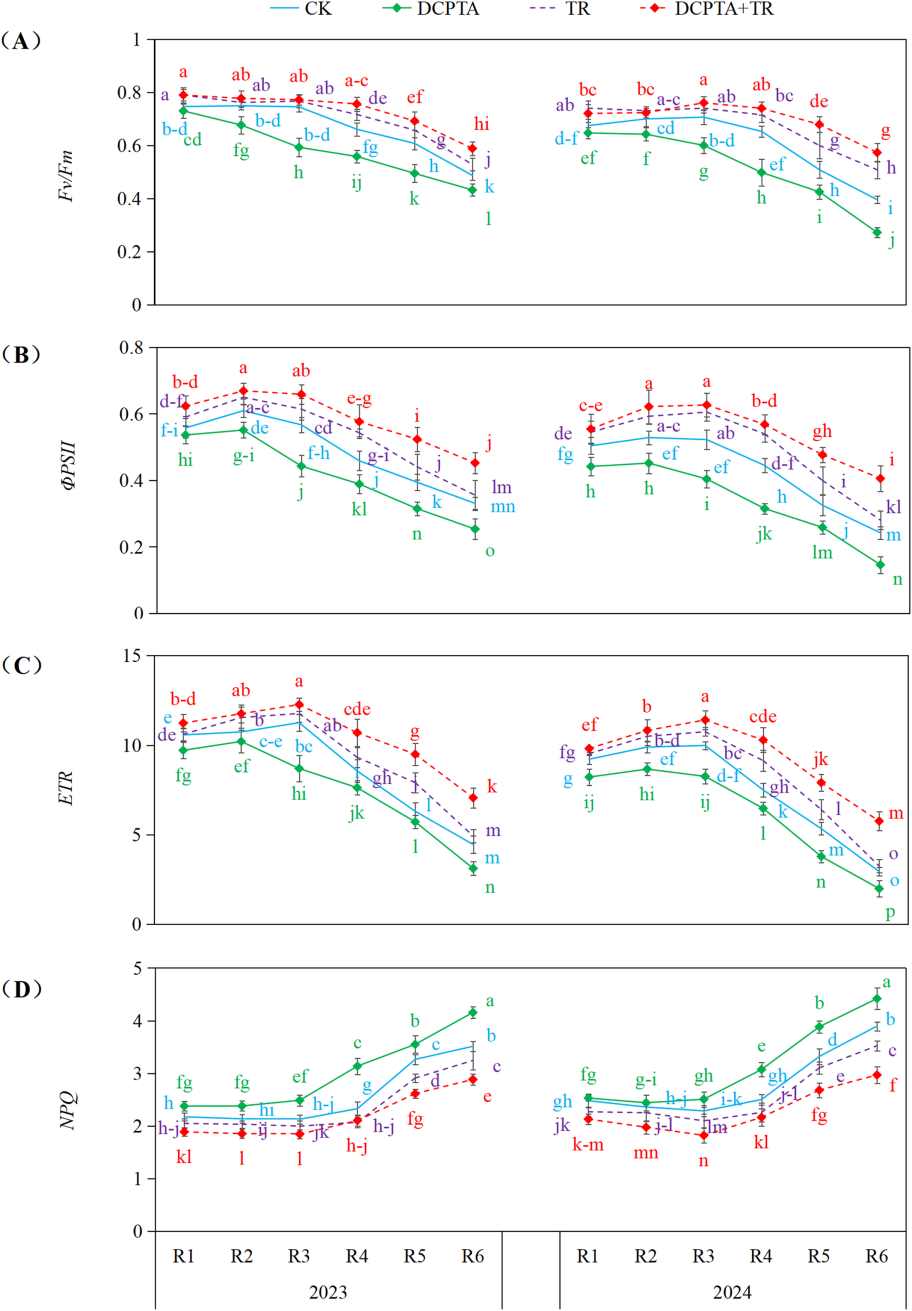
Effects of DCPTA and/or top removal on chlorophyll fluorescence parameters of the 8th leaves across two growing seasons (2023-2024). **(A)** Represents maximum quantum efficiency of photosystem II (Fv/Fm); **(B)** Represents effective quantum yield of PSII (ΦPSII); **(C)** Represents electron transport rate (ETR); **(D)** Represents non-photochemical quenching (NPQ). Lowercase letters (a, b, c...) indicate significant differences among treatments (P<0.05, LSD test).

Analysis revealed significant impacts of treatments on photosynthetic efficiency (**Figure 6**). DCPTA pretreatment alone consistently impaired performance, significantly decreasing *Fv/Fm*, *ΦPSII*, and *ETR* compared to CK (e.g., 2024 R6: *Fv/Fm* -31.24%, *ΦPSII* -39.76%, *ETR* reductions >20%), while concurrently increasing *NPQ* (peak +34.79%). TR alone generally enhanced photosynthetic parameters (e.g., 2024 R6: *Fv/Fm* + 28.49%, *ΦPSII* + 15.76%, *ETR* + 10.54%) and decreased *NPQ*. Most profoundly, the DCPTA+TR combination not only counteracted the negative effects of DCPTA but induced substantial synergistic increases in *Fv/Fm*, *ΦPSII*, and *ETR*, particularly pronounced during R4-R6. Striking enhancements were observed in 2024 R6 (*Fv/Fm* + 44.91%, *ΦPSII* + 67.45%, *ETR* + 96.14%), accompanied by significant *NPQ* reductions (up to -23.84%).

### 3.8 Gas exchange parameters

**Figure 7:**
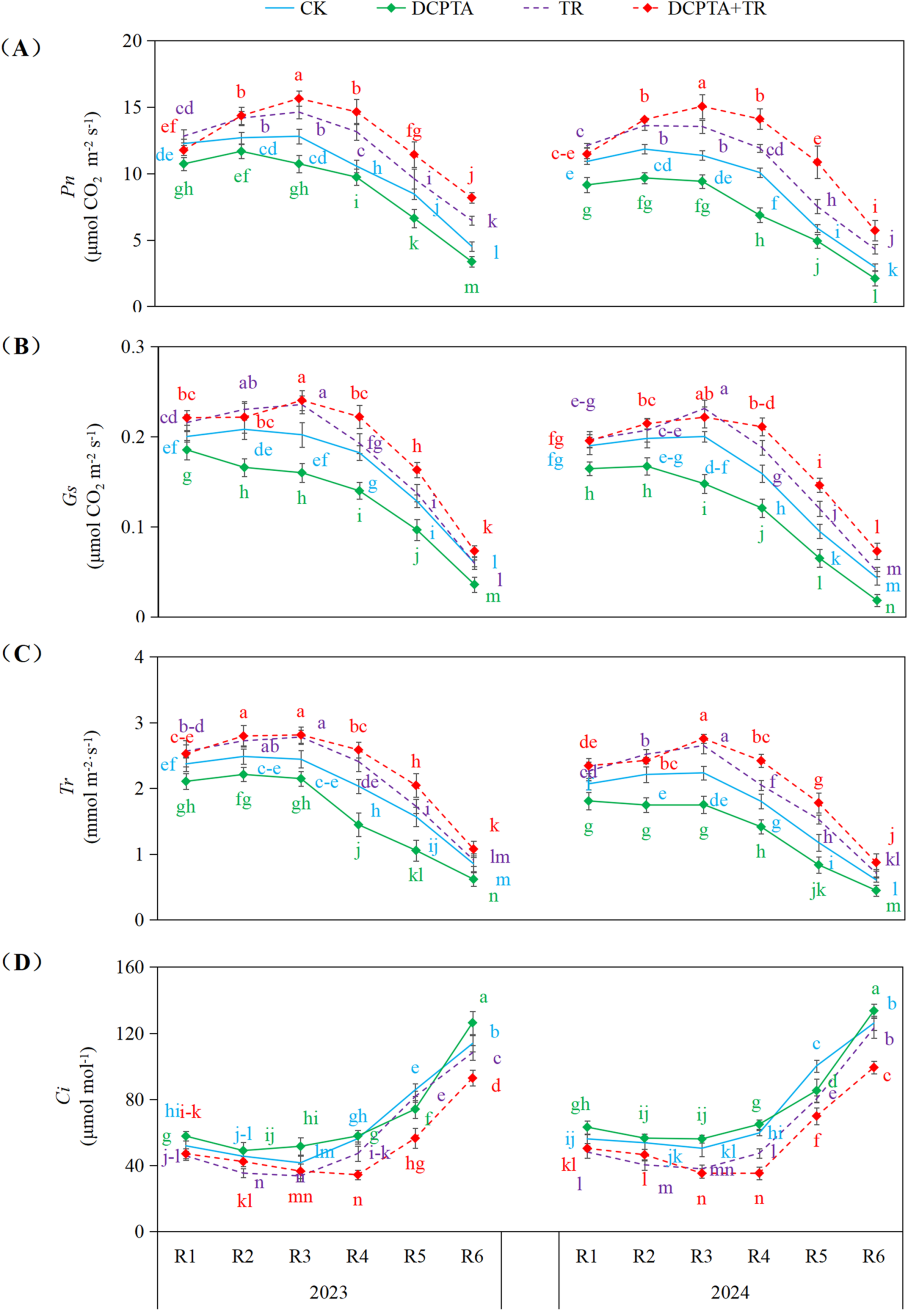
Effects of DCPTA and/or top removal on gas exchange parameters of the 8th leaves across two growing seasons (2023-2024). **(A)** Represents net photosynthesis rate (Pn); **(B)** Represents stomatal conductance (Gs); **(C)** Represents transpiration rate (Tr); **(D)**Represents intercellular CO₂ concentration (Ci). Lowercase letters (a, b, c...) indicate significant differences among treatments (P<0.05, LSD test).

Analysis over consecutive years revealed consistent treatment effects (**Figure 7**). DCPTA pretreatment alone exerted a consistent inhibitory effect on *Pn*, *Gs*, *Tr* across all stages (R1-R6) in both years (e.g., *Pn* reductions often >20% by R5-R6). TR alone consistently enhanced *Pn*, *Gs* (except 2023 R6), and *Tr*, with *Pn* increases peaking at later stages (e.g., 2024 R6: +46.08%). Critically, the DCPTA+TR combination demonstrated potent synergy. While variable early (R1-R3), it induced dramatic, progressive enhancements in *Pn*, *Gs*, and *Tr* from R4 onwards, culminating in exceptionally high *Pn* increases at R5-R6 (e.g., 2024: +84.63% to +93.57%). This synergy was paralleled by significant reductions in *Ci* under the combination, particularly at R4-R6 (down to -40.68%), indicating enhanced carbon assimilation efficiency. The magnitude of synergistic enhancement was generally greater in 2024.

### 3.9 Carbon assimilation enzyme activities

**Figure 8:**
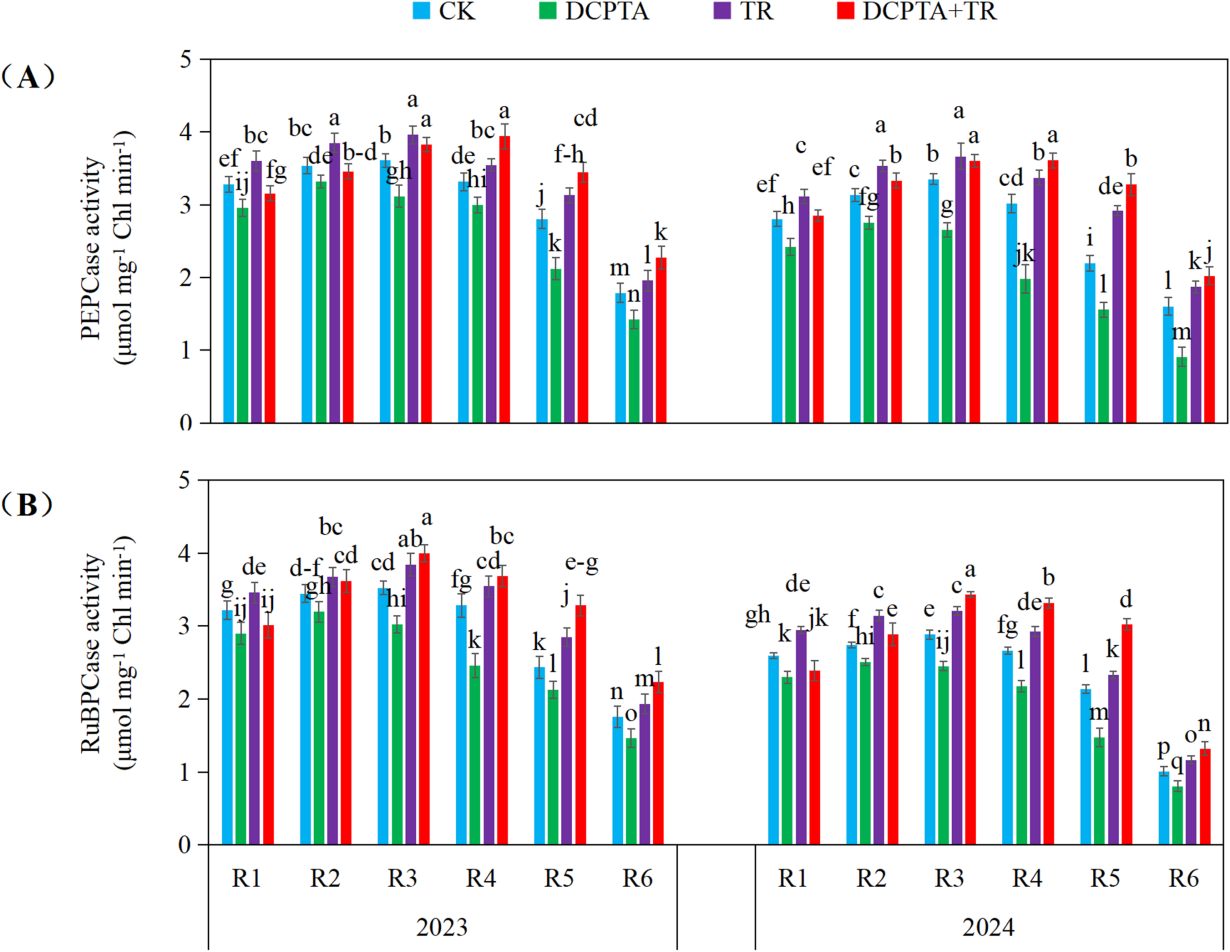
Effects of DCPTA and/or top removal on carbon assimilation enzyme activities of the 8th leaves across two growing seasons (2023-2024). **(A)** Represents phosphoenolpyruvate carboxylase (PEPCase) activity; **(B)** Represents ribulose-1,5-bisphosphate carboxylase/oxygenase (RuBPCase) activity. Lowercase letters (a, b, c...) indicate significant differences among treatments (P<0.05, LSD test).

Comprehensive analysis revealed distinct impacts on PEPCase and RuBPCase activities (**Figure 8**). DCPTA pretreatment alone consistently suppressed both enzymes across all stages (R1-R6) in both years, with suppression intensifying in 2024 (e.g., R6 PEPCase: -43.28%; R5 RuBPCase: -30.97%). TR alone consistently enhanced both enzymes annually (e.g., 2024 R5 PEPCase: +32.91%; 2024 R1-R2 RuBPCase: +13.60%, +14.78%). Crucially, the DCPTA+TR combination demonstrated potent synergy, especially pronounced in later stages (R4-R6) and intensifying in 2024. This treatment not only overcame DCPTA-induced suppression but resulted in substantial activity increases, peaking at 2024 R5 (PEPCase: +49.28%; RuBPCase: +41.62%). The synergistic enhancement exhibited clear time-dependency (stronger in 2024) and stage-progression (maximal at R5/R6).

## 4 Discussion

### 4.1 Resolving the DCPTA paradox through strategic top removal

The well-established yield-enhancing properties of the tertiary amine growth promoter DCPTA across diverse crops, as reported by Gausman et al. (1985); Hartz et al. (1995); Gao et al. (2022), and Li et al. (2025), are corroborated in our study, with grain yield increases of 5.69% (2023) and 4.56% (2024) under treatment (**Table 2**). This benefit may stem mechanistically from DCPTA-driven enhancement of photosynthetic capacity (Li et al., 2020) and modulation of antioxidant system (Xie et al., 2018), translating to greater kernel number per ear and 1000-grain weight. However, this positive effect is intrinsically counterbalanced by a critical vulnerability: DCPTA-induced hyper-proliferation of the upper canopy intensifies intraspecific competition, diverting photosynthates away from basal internode development. The resulting compromise in mechanical strength precipitates significant lodging (**Figure 2**), thereby reducing harvestable ear number and fundamentally capping yield potential—a recognized limitation of growth promoters (Li et al., 2019).

This study confirms that the top removal (TR) treatment effectively resolves the aforementioned trade-off. By removing key photosynthetic sink organs of the plant, this treatment significantly restructures the canopy architecture (consistent with the canopy optimization principle reported by Liu et al., 2015) and thereby produces dual effects: on one hand, it enhances the transmission efficiency of light to the basal leaves and improves the photosynthetic microenvironment of the lower leaves; on the other hand, it redirects assimilates toward structural reinforcement and grain filling. The physiological improvements induced by the TR treatment are further validated: compared with the control group (CK), the TR treatment significantly reduces the incidence of maize lodging (P<0.05); for plants that do not lodge, the TR treatment also outperforms the CK treatment in improving stem plumpness and grain plumpness.

While the yield gain from TR treatment alone is limited, the synergistic effect between TR and DCPTA when applied in combination constitutes a key innovation. This integrated strategy generated supra-additive yield increases of 9.34% (2023) and 8.19% (2024), not only amplifying DCPTA’s beneficial effects on grain characteristics but also effectively counteracting its inherent propensity to induce lodging. This also better aligns with the production requirement of “prioritizing stable yield” in the cold regions of Northeast China. Resolution of this yield-stability trade-off arises from the capacity of top removal to redirect photosynthates, enhanced by DCPTA, towards fortifying the biomechanical integrity of basal internodes.

### 4.2 Metabolic reprogramming underpinning structural synergy

Mechanical strength in basal internodes—paramount for lodging resistance (Kamran et al., 2018)—develops during grain filling via metabolic remodelling (Larson, 2020). Focusing on the critical third basal internode, a biomechanical lever point exhibiting peak susceptibility (Xue et al., 2020), we observed an initial strength increase (stages R1–R2) in controls, followed by progressive decline to mechanical minima at R6. The accelerated decline in *BS* relative to *PS* identifies *BS* as the primary determinant of structural failure (Al-Zube et al., 2018; Sekhon et al., 2020), reflecting resource reallocation to grains and tissue senescence (Wu et al., 2019).

DCPTA application exacerbated this vulnerability by intensifying sink competition, and this suppressing synthesis of critical cell wall components (Yang et al., 2020). Conversely, top removal enhanced internode strength (Xue et al., 2017). Remarkably, the combination treatment induced synergistic mechanical reinforcement beyond additive effects (**Figure 2**). This enhancement is mechanistically rooted in coordinated changes to structural carbohydrate pools (**Figure 3**): Increased cellulose and hemicellulose bolster tensile strength and toughness (Qi et al., 2023), while amplified lignin deposition dramatically augments rigidity and compression resistance (Wang et al., 2024)—both strongly correlated with *PS* and *BS* (Xue et al., 2016).

Crucially, the combination hierarchically activates the phenylpropanoid biosynthetic cascade (**Figure 4**). While DCPTA suppressed PAL (the gateway enzyme initiating carbon flow into lignin biosynthesis; Khobra et al., 2024), top removal (TR) strongly induced PAL activity. Their integration triggered a supra-additive induction of PAL—with 2023 R5 stage PAL activity increasing by 31.74% vs. CK and 18.26% vs. TR alone (**Figure 4**)—which provided more initial catalytic capacity for phenylpropanoid pathway initiation, relative to TR alone (Olsen et al., 2008; Lei et al., 2025). This surge subsequently drove substrate-induced upregulation of 4-CL activity (Sun et al., 2013). Most significantly, the combination not only reversed DCPTA’s suppression of CAD and POD—key enzymes for monolignol synthesis and oxidative cross-linking—but enhanced their activities above control levels. Restored CAD efficiently channels intermediates into polymerizable monolignols, while amplified POD activity drives their oxidative integration into the cell wall matrix (Ma et al., 2024). This concerted enzymatic upregulation culminates in robust lignin polymer deposition and cell wall thickening, delivering the observed mechanical fortification (Hussain et al., 2019).

### 4.3 Reconfigured photosynthetic dynamics fueling structural reinforcement

The synthesis of stalk structural substances depends on continuous photosynthetic carbon supply; thus, we need to further analyze how DCPTA+TR regulates the photosynthetic system to clarify the link between carbon source supply and structural reinforcement. Sustained carbon flux from photosynthesis is fundamental to stalk reinforcement. We dissected this linkage by examining source-sink dynamics: Basal leaves (notably the 8th position) supply assimilates to the developing 3rd internode during grain filling (Fournier and Andrieu, 2000). While top removal augmented *Pn* in basal leaves by improving *PAR* interception, DCPTA alone suppressed it (**Table 2**; **Figure 7**; Xue et al., 2016)—contrasting its stimulatory effect on ear leaves (Xie et al., 2019b) and seedlings (Xie et al., 2017). This suppression may stem from DCPTA-induced canopy expansion, which intensifies shading and degrades light quality in lower strata (Scartazza et al., 2016; Gao et al., 2022). Critically, the combination treatment’s failure to fully reverse DCPTA’s negative impact on basal *Pn* revealed that PAR optimization alone is insufficient; underlying photobiochemical adaptations are essential.

We identified synergistic photophysiological adjustments underpinning resilience in the combination treatment: Preservation of light-harvesting complex integrity and enhanced *de novo* chlorophyll synthesis [49] countered DCPTA’s acceleration of chlorophyll degradation (**Figure 5**; Pedraza-González et al., 2024) and decline in *Fv/Fm* during late grain filling (**Figure 6**; Xu C, et al., 2024). This adaptation facilitated greater photon absorption and electron transport (Nie et al., 2024). Paradoxically, the combination also substantially elevated *NPQ*. This enhanced capacity for thermal dissipation provides critical photoprotection against over-excitation under fluctuating light, safeguarding photosynthetic function (Pan et al., 2018).

Furthermore, stomatal dynamics revealed a fundamental temporal advantage: DCPTA alone accelerated the transition from stomatal to non-stomatal limitations in basal leaves, evidenced by earlier attainment of minimum *Ci* (**Figure 7**; Signarbieux and Feller, 2011). Strikingly, the DCPTA + top removal combination significantly delayed this transition, extending the period of high photosynthetic capacity before biochemical limitations dominate (Wu et al., 2024). Mechanistically, this delay correlated with sustained activity of carboxylation enzymes (PEPC, Rubisco) and moderated decline in *Gs* (**Figures 7**, **8**). Consequently, the combination uniquely extends the functional photosynthetic window, maintaining elevated carbon fixation longer into grain filling to fuel stalk reinforcement.

## 5 Conclusion

This work establishes that strategic integration of foliar-applied DCPTA (35 mg L^-1^ at V8) and mechanical top removal (tassel + 2 uppermost leaves at R1) simultaneously maximizes grain yield and lodging resistance in maize—resolving a persistent trade-off in plant growth regulator application. Top removal reconfigures canopy architecture, mitigating DCPTA-induced shading of critical basal source leaves and redirecting assimilates toward structural sinks. The enhanced carbohydrate flux into the pivotal basal internode, potentiated by DCPTA, supra-additively activates the phenylpropanoid pathway through hierarchical upregulation of PAL, 4-CL, CAD, and POD. This metabolic reprogramming drives robust deposition of lignin, cellulose, and hemicellulose, dramatically strengthening the stalk’s biomechanical core. Consequently, the combined treatment reduces lodging while synergistically enhancing kernel number, kernel weight, and harvestable ear density, yielding grain increases with superior stability. The two-year average grain yield increased by 8.77% compared with CK and 5.10% compared with the DCPTA-only treatment.

This sink-managed metabolic optimization paradigm offers a scalable conceptual guide that may inform sustainable crop intensification efforts. However, note that this conclusion applies to the tested conditions: maize hybrid ‘Zhengdan 958’ grown in a temperate continental monsoon climate (Harbin, China; 45°46′N, 126°54′E) with 18–22°C growing-season mean temperature, 450–550 mm rainfall, neutral loam soil (pH 7.2–7.3), 7 plants·m^−2^ density, and basal slow-release fertilizer (25% N, 10% P_2_O_5_, 13% K_2_O) at 700 kg·ha^−1^. Efficacy may decrease for other genotypes, climates, soils, or management practices, requiring further validation for broader use.

## Data Availability

The original contributions presented in the study are included in the article/**Supplementary Material**. Further inquiries can be directed to the corresponding author.
